# High genetic burden of type 2 diabetes can promote the high prevalence of disease: a longitudinal cohort study in Iran

**DOI:** 10.1038/s41598-020-70725-4

**Published:** 2020-08-19

**Authors:** Maryam Moazzam-Jazi, Leila Najd Hassan Bonab, Asiyeh Sadat Zahedi, Maryam S. Daneshpour

**Affiliations:** Cellular and Molecular Endocrine Research Center, Research Institute for Endocrine Sciences, Shahid Beheshti University of Medical Sciences, Tehran, Iran

**Keywords:** Genetics, Diseases, Medical research, Molecular medicine

## Abstract

Type 2 diabetes (T2D) is emerging as one of the serious public health issues in both developed and developing counties. Here, we surveyed the worldwide population differentiation in T2D-associated variants and assessed the genetic burden of the disease in an ongoing Tehran Cardio-Metabolic Genetic Study (TCGS) cohort represented the Iranian population. We found multiple SNPs that were significantly depleted or enriched in at least one of the five populations of 1,000 Genome Project (African, American, East Asian, European, and South Asian) as well as the Iranian population. Interestingly, *TCF7L2*, a well-known associated gene with T2D, harbors the highest number of enriched risk alleles almost in all populations except for East Asian, where this gene embraces the largest number of significantly depleted risk alleles. The polygenic risk score (PRS) of the enriched risk alleles was calculated for 1,867 diabetic and 2,855 non-diabetic participants in the TCGS cohort, interestingly demonstrating that the risk of developing T2D was almost two times higher in top PRS quintile compared with the lowest quintile after adjusting for other known risk factors.

## Introduction

Type 2 diabetes (T2D) is one of the major life-threatening diseases globally, accounting for 4.2 million deaths worldwide in 2019 as assessed by International Diabetes Federation (IDF) consortium^1,2^. According to the IDF report, among 20 countries belongs to the Middle East and North Africa (MENA) region, Iran is ranked third with the highest number of adults (5.4 million) who suffered from diabetes. The prevalence of diabetes in Iran's adult population was 11.4% in 2014, estimating 9.2 million Iranian individuals will have diabetes by the year 2030^3^.

Type 2 diabetes is a common multifactorial metabolic disease, resulting from both genetic and non-genetic (environmental) factors. The heritability of T2D ranges from 20 to 80%, suggesting the considerable role of genetic factors in the development of T2D; the heritable component of the disease is polygenic where many genes and their variants contribute to an enhanced risk of T2D development^4^. The advent of high-throughput genotyping technologies has created a significant breakthrough in understanding the underlying genetic components of complex diseases, including T2D. A large number of common and low-frequency T2D susceptibility variants have been characterized by the genome-wide association studies (GWAS) and the whole-genome sequencing^5–9^. Most of these variants are located near genes that were previously known to be involved in diabetes pathogenesis, such as *TCF7L2, CDKAL1, CDKN1C,* and *IGF2BP2*^10^. Among them, *TCF7L2* is responsible for the largest proportion of the T2D-associated variance in the various ethnic groups^11^. *TCF7L2* encodes a transcription factor played a central role in the Wnt signaling pathway to regulate glucose homeostasis^12^. Since not all individuals are equally affected by type 2 diabetes through the unhealthy lifestyle and some are more sensitive than others, the corresponding genetic variants can lead to the population disparities in the T2D prevalence^12,13^.

According to the examination of 16 SNPs, Corona et al. proposed that genetic susceptibility to type 2 diabetes is lower for East Asia and American populations than Africa and Europe populations^14^. Similarly, the population differentiation in the obesity-associated variants and the consequent obesity prevalence was reported^15^. Nowadays, the availability of population-scale disease-related genetic variants has enabled the researchers to survey the variant frequencies across different populations and estimate the genetic burden of disease. A recent study demonstrated that the high number of deleterious variants attributed to the cardiovascular diseases in Pakistan population can cause the increased mutation load of this disease in the population^16^. Considering the complex diseases influenced by many common genetic variants with the small effect size, the meaningful risk assessment requires inspecting the cumulative effect of multiple variants, acquiring with calculating the polygenetic risk score (PRS). Merino et al. computed the polygenic risk score for a large number of diabetic and non-diabetic participants using 68 risk T2D SNPs and indicated that the corresponding genetic burden and the dietary fat quality are associated with the risk of type 2 diabetes^17^. Recognizing and targeting the individuals at the highest genetic risk can increase the cost-effectiveness of lifestyle and other interventions to prevent or delay the T2D incidence^12,18^. The critical importance of the issue in society public health and the early-mentioned statistics regarding the T2D status in Iran motivated us to estimate the genetic burden of this disease in the Iranian population for the first time.

The present study aims to assess the genetic burden of type 2 diabetes in the Iranian population. To this end, (1) we collected a comprehensive list of T2D-associated SNPs and examined their allele frequencies differences between the Iranian population and each of 1,000 Genome Project populations, African (AFR), American (AMR), East Asian (EAS), European (EUR), and South Asian (SAS); (2) we identified the enriched or depleted risk alleles of T2D-associated SNPs in the Iranian and each of the 1,000 Genome Project populations compared to the global population; (3) finally, we calculated the polygenic risk score (PRS) for the enriched risk alleles within the Iranian population and tested the PRS correlation with T2D prevalence and incidence in this population.

## Results

A total of 2,302 T2D-associated SNPs with the genome-wide p-value threshold of 5 × 10^–8^ was selected from NHGRI-EBI GWAS Catalog^19^ and Type 2 Diabetes Knowledge Portal^20^ (Supplementary File 1). The collected SNPs resulted from 21 GWA studies, which two studies conducted in African populations, three in East Asian populations, 12 in European, two in South Asians, and three in American populations. According to the annotation results obtained from the VEP tool, 2,032 of 2,302 SNPs distributed on 163 genes, which 61% of them located in intronic regions (Supplementary File 1); therefore, they most likely affect the transcription regulation of T2D-related genes rather than the gene function.

**Figure 1:**
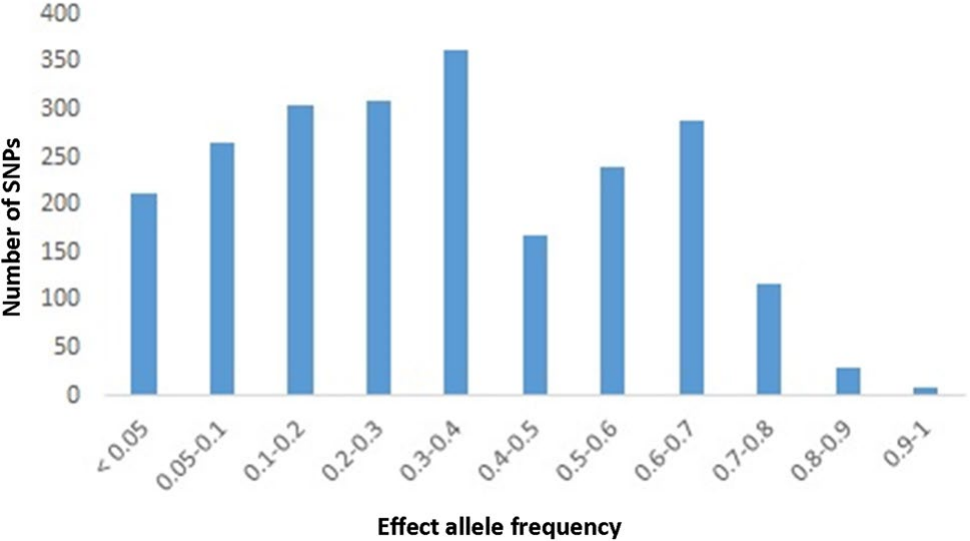
Effect allele frequency spectrum of type 2 diabetes-associated SNPs in Iranian population.

**Figure 2:**
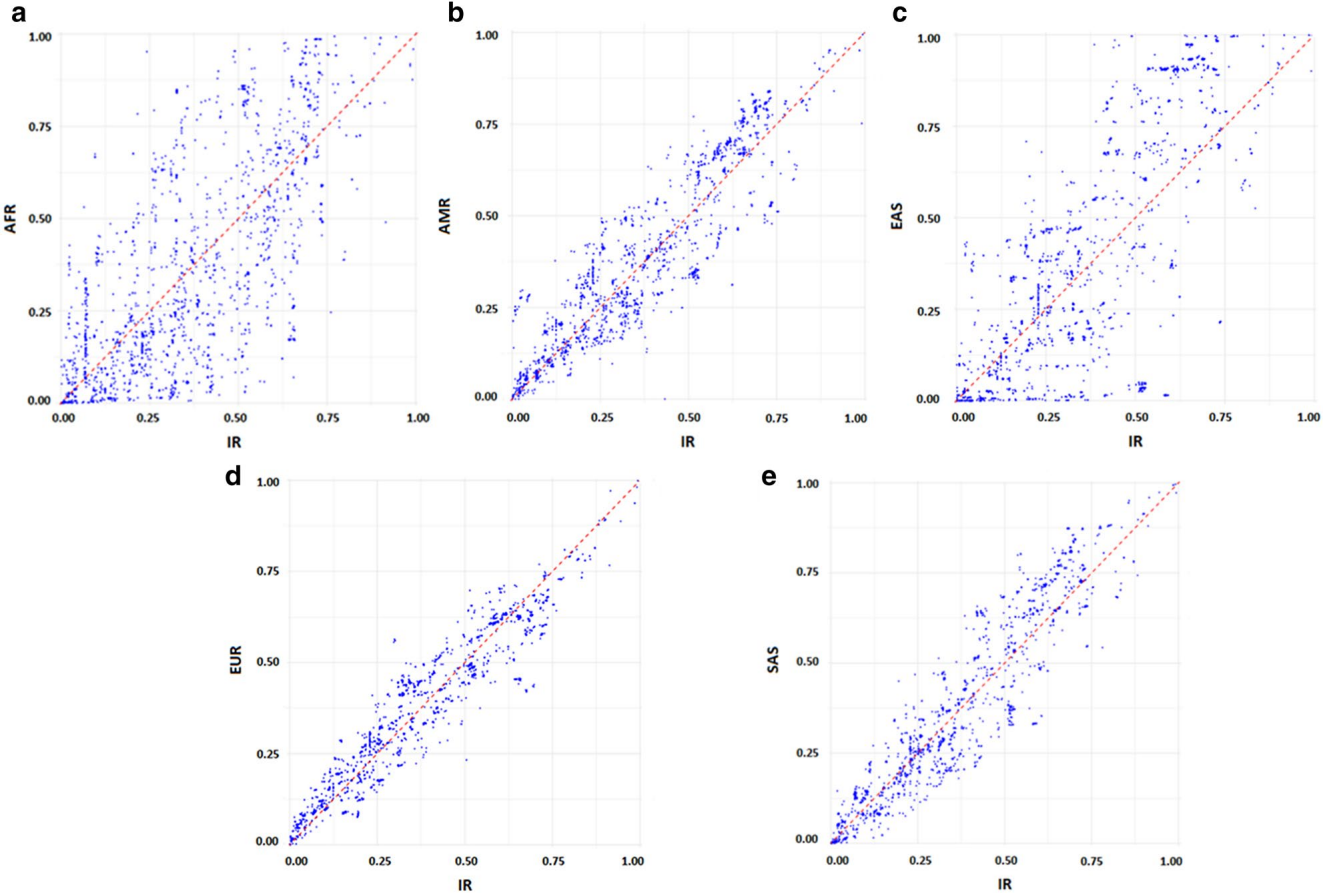
The comparative distribution of effect allele frequencies in Iranian population versus all five populations of 1,000 Genomes Project.

We then acquired the effect allele frequency of selected SNPs in each of five populations of the 1,000 Genomes Project as well as the IR population (whole-genome sequencing data of TCGS cohort) (Supplementary File 2). The effect allele frequency spectrum of all T2D-related SNPs in the Iranian population revealed the distribution of SNPs is skewed toward the higher allele frequencies (AF > 5%). Although the proportion of rare and low-frequent variants were found to be 211 out of 2,302 variants in our population (Fig. 1), the more low-frequent and rarer variants may be detected within the larger sample size. The effect allele frequencies of all SNPs were compared between Iranian and each of five 1,000 Genome Project populations as illustrated in Fig. 2; based on this comparative analysis, a large number of SNPs have a higher effect allele frequency in the Iranian population than the 1,000 genome populations. The maximum difference of effect allele frequency with AMR, EUR, and SAS populations were 3.7, 2.9, and 22.3 times higher in IR, respectively. However, the largest differences in effect allele frequency were detected between IR and EAS as well as between IR and AFR populations where the maximum effect allele frequency difference with these populations was 315 and 79 times higher in IR (Fig. 2 and Supplementary File 2), all of these variants were risk variant. Moreover, the average frequency of T2D risk variants was 0.39 in the Iranian population, consistent with the general population and the natural selection impact to keep fitness-reducing alleles at low frequency^4,21^.

### Genetic population differentiation

**Figure 3:**
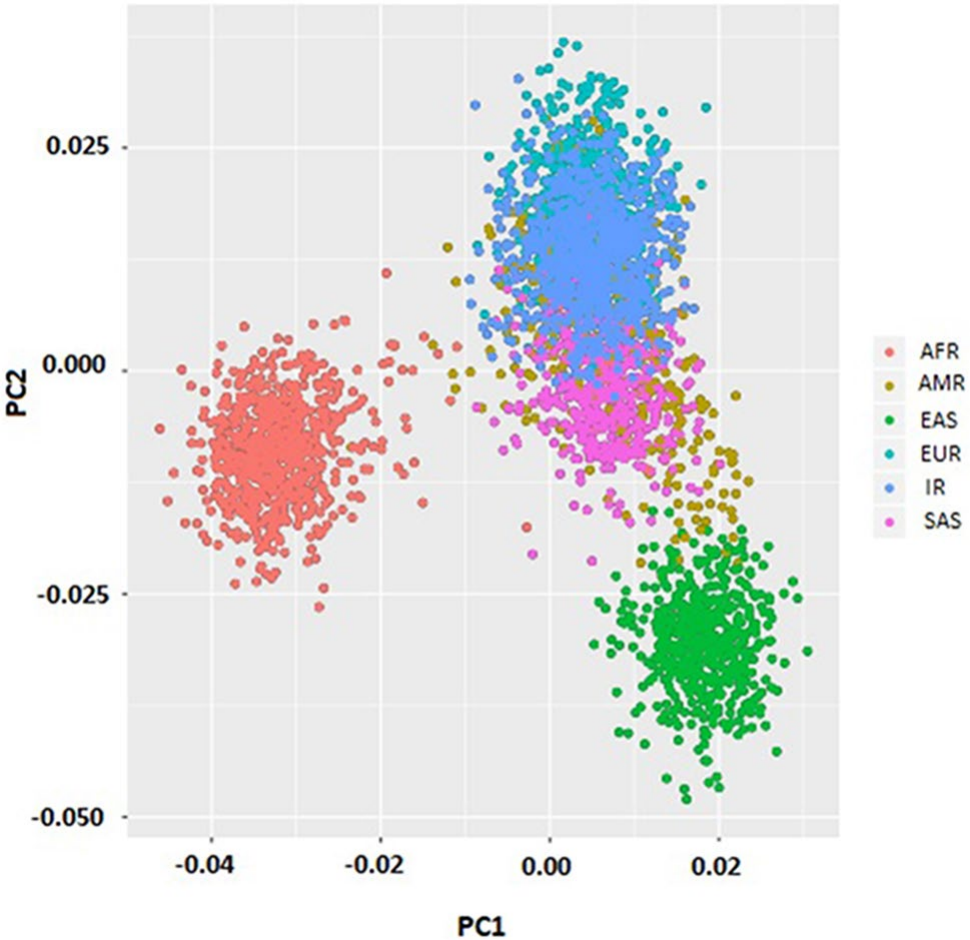
Principal Components Analysis (PCA) based on type 2 diabetes-related SNPs.

In the present study, the population differentiation of all SNPs was also determined through calculating the Weir and Cockerham Fst for the IR population versus each of five populations in the 1,000 Genomes Project. The mean Fst value for IR population against SAS and EUR populations were 0.018509 and 0.01134, respectively while it was found to be 0.02529 against AMR population; interestingly, the EAS and AFR populations displayed the largest differentiation where the means Fst value of 0.091127 and 0.086663 were computed for these populations, respectively, against the IR population. Additionally, there are 109 SNPs with high Fst value (Fst > 0.05) out of 2,302 T2D-associated SNPs, ranking above the top 1% across all populations (AFR, AMR, EAS, EUR, IR, and SAS). As the SNP with the high Fst value can specify the positive selection and/or population expansion, we also performed the same Fst analysis for the randomly selected SNPs between IR and each of five populations of 1,000 Genome Project and recognized 120 out of 2,302 random SNPs ranked above top 1% among all populations. This frequency difference was not significant (Chi^2^ = 0.178, p-value = 0.67), suggesting there is no clue of positive selection at T2D-linked SNPs compared with the random SNPs in the genome. The observed differences in allele frequencies and the Fst value of SNPs of interest provided a sign of global population stratification based on the T2D-related SNPs. Therefore, the principal component analysis was also conducted for the variants within the populations under study. As Fig. 3 illustrated, all populations clustered together except for African and East Asian locating at the distinct groups, consistent with the high mean Fst values compared with other populations (Fig. 3). Further details on PCA analysis can be available at the Supplementary File 3.

### Enrichment analysis of T2D-associated SNPs

With the above information in mind, we examined which risk allele of T2D-associated SNPs is enriched or depleted in each population (AFR, AMR, EAS, EUR, SAS, and IR) in comparison with the global population. Based on our results, 197 out of 212 risk SNPs were significantly depleted or enriched at the FDR threshold of 0.05 in at least one of six populations. The significantly altered SNPs were distributed on 15 genes. We further investigated the top four genes with the highest number of enriched or depleted risk SNPs in the various populations (Table 1), which *TCF7L2* harbors the highest number of enriched risk SNPs in Iran (62 SNPs), African (77 SNPs), and European (63 SNPs) populations while this gene was ranked as one the top four genes carrying the largest number of both enriched and depleted SNPs in AMR and SAS populations. Interestingly, the EAS population revealed a different pattern where no *TCF7L2* SNP significantly enriched in this population; instead, we found 75 significantly depleted risk SNPs of this gene in the individuals with EAS ancestry. Correspondingly, *CDKAL1* was found to be the gene with the highest number of enriched and the lowest number of depleted risk variants in the EAS population whereas a large number of risk SNPs of *CDKAL1* was significantly both enriched and depleted in AFR, AMR and SAS populations. However, IR and EUR populations presented a distinct pattern where *CDKAL1* was ranked as one of the top genes with the highest number of depleted risk variants in these populations (Table 1).

**Table 1:** Top four genes carrying the highest number of enriched and depleted T2D-associated SNPs in various populations.

	IR		AFR		AMR		EUR		SAS		EAS	
	Gene	Count	Gene	Count	Gene	Count	Gene	Count	Gene	Count	Gene	Count
Enriched	*TCF7L2*	62	*TCF7L2*	77	*TCF7L2*	24	*TCF7L2*	63	*TCF7L2*	28	*CDKAL1*	30
	*CDKAL1*	4	*CDKAL1*	33	*SLC16A11*	19	*CDKN1C*	4	*IGF2BP2*	14	*SLC16A11*	13
	*CDKN1C*	4	*IGF2BP2*	31	*SLC16A13*	3	*CDKAL1*	1	*CDKN1C*	4	*CDKN2B*	2
	*TECRL*	1	*SLC16A11*	19	*HLA-DQA2*	1	*TECRL*	1	*CDKAL1*	1	*SLC16A13*	2
Depleted	*IGF2BP2*	30	*TCF7L2*	30	*IGF2BP2*	30	*IGF2BP2*	31	*TCF7L2*	32	*TCF7L2*	75
	*SLC16A11*	18	*CDKAL1*	18	*TCF7L2*	30	*CDKAL1*	26	*SLC16A11*	19	*IGF2BP2*	30
	*CDKAL1*	16	*SLC16A11*	12	*CDKAL1*	29	*SLC16A11*	17	*CDKAL1*	17	*CDKN1C*	4
	*TCF7L2*	3	*CDKN1C*	2	*CDKN1C*	3	*SLC16A13*	3	*SLC16A13*	3	*SLC16A11*	4

### *TCF7L2* SNPs

**Figure 4:**
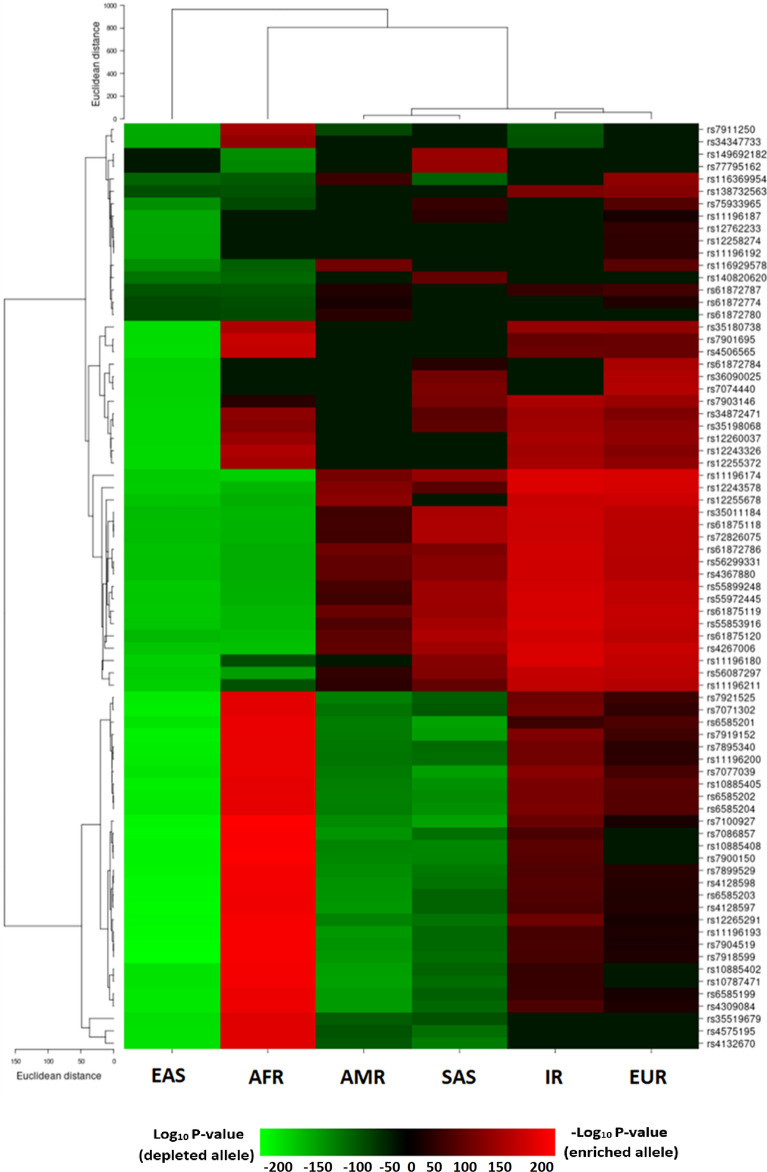
Heatmap illustrates the significantly enriched or depleted risk alleles of *TCF7L2* SNPs in each population. Each row and each column represent the SNP and the population, respectively.

The prominent contribution of *TCF7L2* variants in the risk of developing type 2 diabetes as well as a large number of significantly enriched SNPs of this gene in the Iranian population compared to the global population motivated us to further interrogate the *TCF7L2* variants enrichment/depletion pattern in all populations. The heatmap illustrated how significantly the risk alleles of *TCF7L2* SNPs were depleted or enriched across populations (Fig. 4). Based on our enrichment analysis, a total of 77 risk alleles of *TCF7L2* were enriched or depleted in at least one of the populations of interest. As Fig. 4 displayed, these 77 SNPs were clustered into two major groups, one group consisting of 48 SNPs and other group consisting of 29 SNPs, which exhibit discrete allele enrichment/depletion patterns. In the 48-member group, the risk allele of all *TCF7L2* SNPs, except for one or two SNPs, was enriched in AMR, EUR, IR, and SAS populations while except for 10 enriched risk alleles in AFR population, these risk alleles were depleted in individuals with EAS and AFR ancestries. Similarly, in the 29-member group, the risk alleles of *TCF7L2* SNPs were enriched in AFR, IR, and EUR populations, but depleted in individuals with EAS, SAS, and AMR ancestries (Fig. 4).

### Polygenic risk score analysis and its association with prevalent T2D

In addition to genotype data, the phenotype data including the T2D status and some blood biochemical parameters were available for the Iranian population (TCGS cohort); it is worth to note that we used the unrelated individuals with the available phenotype data and genotype data obtained by chip-typing or imputing for this part of the study. Here, we surveyed if there is any correlation between the enriched T2D-associated risk SNPs and the type 2 diabetes incidence in Iran. To this end, the cumulative effect of enriched risk SNPs on T2D disease was examined by calculating the polygenic risk score, which combined the individual genetic effects into a single measure. The weighted PRS was computed based on the enriched risk SNPs for 4,722 individuals aged 20 and older (1,867 diabetic and 2,855 non-diabetic individuals) using the PRSice tool. Due to the relatively high prevalence of T2D in the population, the robust Poisson regression analysis was utilized to assess the association of computed polygenic risk score with the prevalent T2D^22^. Based on our results, there is a significant association between the PRS and the observed T2D prevalence. As Table 2 indicates, interestingly, the estimated prevalence ratio (PR) was increased with increasing the PRS quintiles in both unadjusted and adjusted models for age, sex, and BMI; however, the statistically significant association (p-value < 0.05) was observed between the top PRS quintile (4th and 5th quintiles) and prevalent T2D. So that, the PR for the genetically high-risk individuals who located in the 4th and 5th quintiles were 1.44 (95% CI 1.2–1.7) and 1.34 (95% CI 1.14–1.6), respectively, compared to the genetically low-risk individuals who located in the bottom PRS quintiles in the age-, sex-, and BMI-adjusted model (Table 2).

**Table 2:** The association of PRS with the prevalence of type 2 diabetes.

Quintile	Sample size	PR (95% CI)	p-value
2 (20–40%)	713	0.9 (0.76, 1.1)	0.53
3 (40–60%)	638	1.05 (0.86, 1.2)	0.6
4 (60–80%)	826	1.34 (1.1, 1.6)	0.001
5 (> 80%)	631	1.44 (1.2, 1.7)	0.00006

Prevalence ratio (PR) with 95% confidence intervals (95% CI) from robust Poisson regression analysis. The model is adjusted for age, sex, and BMI. The first quintile (< 20%) containing 766 individuals were considered as reference. The whole sample size was 3,574 individuals.

### Validation of the PRS with incident T2D analysis

**Figure 5:**
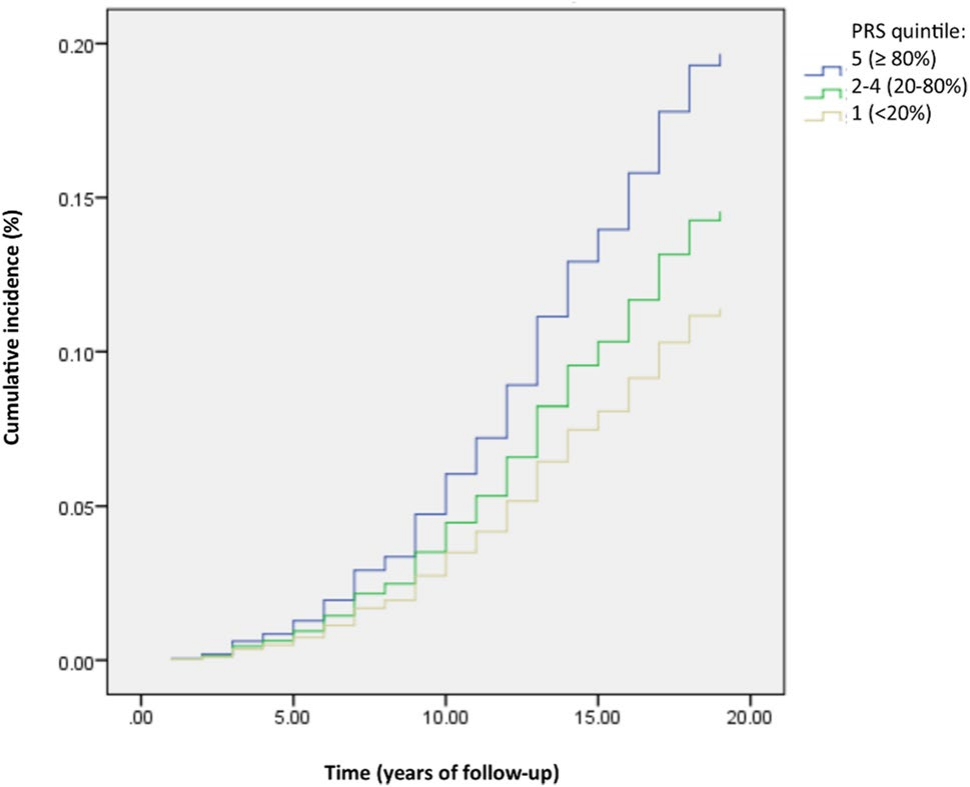
The cumulative incidence of type 2 diabetes in 4,003 genotyped individuals free of T2D at baseline. Cumulative incidence presented separately in three low, moderate and high genetically risk categories.

As the TCGS data resulted from a prospective cohort study, we took advantage of the Cox regression analysis to evaluate the hazard ratios (HRs) and 95% confidence intervals (CIs) for the risk of developing T2D in each PRS quintile. As indicated in Table 3, PRS had a significant effect on the risk of T2D development during the follow-up; both, T2D risk development and PRS quintile followed a similar pattern so that the increased risk of developing T2D was noticed with the increased PRS quintile. The risk difference between the lowest and highest PRS quintiles was about twofold (HR = 1.96, 95% CI 1.4–2.5, p-value = 0.00001) in the adjusted model for age, sex, BMI (Table 3). Interestingly, when the effect of PRS on T2D incidence was determined with additionally adjusting the model for fasting plasma glucose, 2-h plasma glucose, cholesterol, triglyceride, high-density lipoprotein cholesterol levels, as well as low-density lipoprotein cholesterol level, the estimated HR in the highest PRS quintile compared to the lowest quintile was 1.72 (95% CI 1.4–2.3; p-value = 0.001) (Table 3). Besides, the cumulative incidence graph illustrates the difference in the cumulative T2D incidence among the individuals classified as “low”, “moderate”, and “high” genetic risk category (Fig. 5).

**Table 3:** The PRS effect on the risk of developing type 2 diabetes.

Quintile	Sample size	Age, sex and BMI-adjusted model		Full model	
		HR (95% CI)	p-value	HR (95% CI)	p-value
2 (20–40%)	767	0.9 (0.6, 1.2)	0.53	0.93 (0.6, 1.3)	0.7
3 (40–60%)	726	1.35 (1, 1.8)	0.04	1.32 (0.96, 1.8)	0.08
4 (60–80%)	902	1.5 (1.1, 2)	0.003	1.45 (1.07, 1.9)	0.01
5 (> 80%)	825	1.96 (1.4, 2.5)	0.00001	1.72 (1.2, 2.3)	0. 001

Hazard ratio (HR) with 95% confidence intervals (95% CI) from Cox regression analysis. The full model is adjusted for age, sex, BMI, fasting plasma glucose, 2-h plasma glucose, cholesterol, triglyceride, high-density lipoprotein cholesterol, and low-density lipoprotein cholesterol levels. The first quintile (< 20%) containing 783 individuals were considered as reference. The whole sample size was 4,003 individuals.

### Association of PRS with known biochemical T2D risk factors

In individuals without the prevalent T2D, the effect of PRS on known biochemical risk factors of type 2 diabetes, including fasting blood glucose, 2-h glucose, and cholesterol, triglyceride, and BMI levels was evaluated using the linear regression analysis adjusted for age and sex. Our findings revealed that there is no significant association between the computed PRS and each of these biochemical parameters, except for the fasting plasma glucose level. The fasting plasma glucose content was enhanced with raising the PRS quintile, however, the statistically significant association was observed between the top PRS quintile (fifth) and this T2D biomarker (Supplementary Table 1).

## Discussion

Part of the variability in T2D prevalence across populations can be attributed to the corresponding genetic differences. In the current study, we observed the effect allele frequency of a large number of SNPs varied between Iranian and each of five 1,000 Genome Project populations (Fig. 2). Natural selection or population expansion can modify the allele frequencies among different populations, which can result in the local adaption as well as susceptibility to disease^23^. To further quantify the population differentiation for the T2D-related SNPs, we calculated the pairwise Fst value for all SNPs and noted the highest mean Fst value for IR vs. AFR as well as for IR vs. EAS. However, the mean Fst value and the Fst distribution for the randomly selected SNPs were not significantly different from the T2D-associated SNPs. Accordingly, there is no evidence of positive selection for the T2D-related SNPs, consistent with the previous studies on SNPs linked to obesity and T2D^24,25^. Ayub et al. investigated 65 T2D-associated SNPs in the individuals with various ancestries and reported the positive selection cannot account for the T2D allele frequency differences and the prevalence of the disease in the current populations^24^. However, the signatures of positive selection can be traced using further analyses that are beyond the scope of present study.

The evaluation of significantly enriched or depleted SNPs in a given population through the enrichment analysis is a simple, but powerful approach to visualize the worldwide effect allele frequencies distribution^15^. Our enrichment analysis carried out on the comprehensive set of 212 T2D-associated risk SNPs revealed that the highest number of significantly enriched risk alleles belonged to *TCF7L2* almost in all populations except for the EAS, where this gene harbors the maximum number of significantly depleted risk alleles across populations; several depleted *TCF7L2* risk alleles have also been identified in AFR, AMR, and SAS populations, but only three risk alleles in IR and no risk allele of this gene in EUR populations were depleted. Therefore, the T2D risk allele enrichment/depletion pattern in various populations, specifically in African and East Asian populations is discrete. Analysis of a small set of T2D risk alleles (12 risk alleles) among 11 HapMap populations showed the decreasing allele frequency when humans migrate from Africa to East Asia regions^26^, consistent with the observed pattern of SNPs within the second cluster (29 members) in the current study. Adaptation to the distinct climate and agriculture development across continents during human migration from Africa may give rise to such a difference in T2D risk allele frequency and subsequent genetic predisposition to diabetes^26^. However, the risk alleles of this SNP cluster (the second cluster) enriched in EUR as well as in the IR population, in addition to AFR. Here, a similar genetic pattern appears to be shaped by the corresponding geographic locations. Iran is a substantial resource for human genetic variations in Western Asia^27,28^, where the corresponding genomic variations were shown to have the most similarity to the European variations. Likewise, there is a shared Western Asian ancestry for the Western Asian peoples and early European farmers^28,29^, which may account for the detected similar genetic variations pattern between IR and EUR populations in our study. The risk allele enrichment/depletion patterns of T2D-related SNPs among populations might be beneficial for implementing the population-based interventions and treatments for type 2 diabetes. For instance, with a pharmacogenetics clinical trial, the researchers discovered that the response to both glipizide and metformin (glucose-lowering drugs) in individuals with the risk factors for type 2 diabetes and treatment-naive individuals with the disease has been influenced by T2D-associated *TCF7L2* variants^30^. Considering the significant enrichment of a large number of T2D-associated *TCF7L2* variants in IR or EUR populations, but their depletion in the populations with East Asian ancestry, the drug response and the subsequent treatment efficiency may vary among the different populations.

Here, the significant association of polygenic risk score with T2D incidence can propose the high genetic burden of the enriched risk T2D-related SNPs in the Iranian population as specified by both the robust Poisson regression and the Cox regression analyses. Interestingly, we found that the hazard of T2D incidence differs between the top and bottom PRS quintiles about 2 times and the influence of PRS on the risk of developing T2D is independent of well-known biochemical risk factors including, fasting blood glucose, 2-h glucose, cholesterol, and triglyceride content. Among the biochemical parameters, the observed significant positive association of PRS with only FPG in the individuals with no prevalent T2D can imply that some individuals at high genetic risk might previously show the fasting hyperglycemia and even be pre-diabetic or undiagnosed type 2 diabetic. Furthermore, no significant association of PRS with cholesterol, triglyceride, and BMI levels in these individuals suggested the influence of calculated PRS in the present study on the T2D risk cannot be via its possible effect on the lipid-related traits and obesity. In line with our results, Stancakova et al. reported the significant association of the PRS with FPG and the incidence of type 2 diabetes^31^. Considering the attendance of individuals from the main Iranian ethnic groups living in Iran, including Persians, Azeris, Kurds, Lors, Arabs, Baluchs, Turkmans, Mazanis, and Gilaks at the TCGS cohort, this cohort can be representative of the Iranian population and our obtained results can be attributed to the general Iranian population.

In conclusion, this population-based study indicates the considerable worldwide population differentiation in the risk allele frequencies of type 2 diabetes-related SNPs, which can affect the drug response and the subsequent treatment efficiency in cases with different ancestries. We found that part of the increased prevalence of type 2 diabetes in Iranian population can refer to the high genetic burden of this disease among Iranians as the significant association was detected between the polygenic risk score derived from the enriched risk alleles and the type 2 diabetes incidence in our longitudinal cohort study. Furthermore, we demonstrated the high hazard of T2D development in the genetically high-risk individuals compared to the genetically low-risk individuals when the model adjusted for the well-known predictors, like age, sex, BMI, and other biochemical T2D risk factors. It also implies the appropriate predictive ability of the calculated PRS, which might be useful in the clinical implications.

## Methods

### Subjects and measurements

In this study, Iranian subjects were selected from the TCGS project^32^ that is a part of an ongoing Tehran Lipid and Glucose Study (TLGS) cohort, in which subjects have been genotyped and followed up for cardio-metabolic risk factors every three years since 1999 (1999–2017)^33^. At each visit, written consent was obtained from each subject and referred to trained physicians and laboratories for clinical examinations and blood sampling; in summary, weight and height were recorded using the standard protocols. Body mass index (BMI) was calculated as weight in kilograms divided by height in square meters. Fasting plasma glucose (FPG), 2-h plasma glucose, triglycerides (TG), total cholesterol (TC), high-density lipoprotein cholesterol (HDL-C), and low-density lipoprotein cholesterol (LDL-C) levels were measured according to the standard protocols^34^. Type 2 diabetes was diagnosed based on the fasting plasma glucose ≥ 126 mg/dL or 2-h plasma glucose ≥ 200 mg/dL during an oral glucose tolerance test^35^. The first occurrence of type 2 diabetes in individuals during the follow-up period was considered as diabetic condition^36^. The baseline characteristics of the participants were shown in Table 4.

**Table 4:** Baseline characteristics of the TCGS cohort (1999–2017) participants used for the present study.

Variables	Prevalent T2D cases	Incident T2D cases	Non-progress cases to T2D
Sex (Male/Female, number)	295/424	515/633	1,163/1692
Age (mean ± SD)	53 ± 11	45.5 ± 12	35 ± 12
Body mass index (mean ± SD)	29 ± 4.5	28.97 ± 4.8	25 ± 4.3
Fasting plasma glucose (mean ± SD)	163.57 ± 60	96.95 ± 11.13	84.7 ± 6.5
2-h plasma glucose (mean ± SD)	269.8 ± 89.23	127.38 ± 32.8	91.9 ± 19.5
Cholesterol (mean ± SD)	227.5 ± 48	219.23 ± 44	189 ± 41
High density lipoprotein cholesterol (mean ± SD)	40.52 ± 10	40 ± 9.5	42.56 ± 10.5
Low density lipoprotein cholesterol (mean ± SD)	141.5 ± 38	137.4 ± 34.5	119 ± 33.5
Triglyceride (mean ± SD)	226 ± 111.5	180 ± 75	125 ± 65
Follow-up year (median, IQR)	14 (10–17)		

Moreover, 2,504 individuals from five populations of the 1,000 Genome Project (AFR, AMR, EAS, EUR, and SAS) were utilized^37^. All procedures performed in this study approved by the ethics committee on human subject research at Research Institute for Endocrine Sciences, Shahid Beheshti University of Medical Sciences (code of “IR.SBMU.ENDOCRINE.REC.1395.366”), which were in accordance with the 1964 Helsinki Declaration and its later amendments or comparable ethical standards. Written informed consent was obtained from all participants.

### Genotype data

For Iranian subjects, the whole genome of 1,162 participants at the TCGS cohort was sequenced using Illumina Hiseq platform with the average coverage of 30 × and the multi-sample VCF files were generated using GATK pipeline (unpublished data, available upon request). Additionally, the DNA samples of 13,399 TCGS participants were genotyped with Illumina Human OmniExpress-24-v1-0 bead chip containing 649,932 SNP loci at the deCODE genetics company (Iceland) according to manufacturer’s specifications (Illumina Inc., San Diego, CA, USA)^32^. Furthermore, the imputation based on the available whole-genome sequencing data was performed to fill the missing (un-genotyped) variants in the SNP array. The relevant quality control steps were considered for producing high-quality variants at each stage. For the current study, two different datasets were applied. (1) The genotype data of 842 unrelated and non-diabetic individuals derived from whole-genome sequencing was used as VCF format for the comparative effect allele frequency of T2D-related SNPs and enrichment analysis. (2) The genotype data of 4,722 individuals, aged from 20 to 80 years, diabetic (1,867 individuals) and non-diabetic (2,855 individuals) acquired by chip-typing or imputing was used as PLINK format for calculating the polygenic risk score and inspecting its association with T2D incidence in the Iranian population. The pre-diabetic individuals were not considered in this study.

Phase 3 genotype data of the 1,000 Genome Project (VCF files) was downloaded from 1,000 Genome Project website^37^^.^ The variant coordinates were based on the human genome assembly GRCh38.

### Preparation of SNP list

The T2D-associated SNPs were obtained from NHGRI-EBI GWAS Catalog^38^ as well as the comprehensive T2D Knowledge Portal^20^ with the genome-wide p-value threshold of 5e-8. The type 2 diabetes risk alleles of selected SNPs were determined by examining the sign of odds ratio (OR); here, the OR of equal to or greater than 1.2 was considered as the risk allele. Additionally, the orientation of effect (risk) alleles (forward or reverse strand) was checked and the effect (risk) alleles converted to its complement in the case of reporting on the reverse strand.

### Annotation of the selected SNPs

The Variant Effect Predictor (VEP, release 96) tool was used to annotate the selected SNPs^39^ through Ensembl/GENCODE and RefSeq transcripts database.

### Comparison of the variants across worldwide populations

The effect allele frequencies of selected T2D-associated SNPs were obtained from the corresponding VCF files using bcftools (1.9.1). The effect allele frequencies of the variants in the Iranian population were compared with all five major populations of the 1,000 Genome Project. Further, to specify the population-wise genetic differentiation for type 2 diabetes, the pairwise Weir and Cockerham Fst^40^ values were calculated for the available genotype data using VCFtools (v.0.1.15)^41^. To examine if the Fst values of selected T2D-related SNPs were significantly different from that of random SNPs, we selected 2,302 random SNPs with similar allele frequency to the T2D-associated SNPs and calculated the pairwise Fst for these random SNPs. The Fst distribution SNP with high Fst value (Fst > 0.05) of the random SNPs and the T2D-associated SNPs was compared via Chi-Square test^25^. Additionally, the Principal component analysis (PCA) was performed to infer the population structure based on the T2D-associated SNPs using R Package SNPRelate^42^.

### Enrichment analysis of T2D-associated variants

The Fisher’s exact test (implemented in R statistical package) was used to test whether the risk allele of T2D-associated SNPs is significantly enriched or depleted in each of six populations (AFR, AMR, EAS, IR, EUR, and SAS) compared to the global population, which encompasses all six populations together. The false discovery rate (FDR) cutoff of 0.05 was considered significant. For drawing heatmap to visualize the pattern of the allele enrichment/depletion across populations, the obtained FDR was transformed to log_10_. The negative of log_10_ of FDR (a positive number) was used in the case of allele enrichment in a population to show the related SNP in that population in the heatmap. Similarly, if the risk allele of a SNP is depleted in a population, the log_10_ of FDR (a negative number) was applied to display the SNP in that population in the heatmap. The heatmap was generated using R (v.3.6)^43^.

### Polygenic risk score calculation and its association with the prevalence of T2D in Iranian population

We computed the polygenic risk score for all participants in the TCGS cohort aged 20 and older (20–80 years old), composing of 1867 diabetic and 2,855 non-diabetic individuals using PRSice software (v.2.1.6)^44^; the pre-diabetic individuals were excluded from the analysis. PRSice calculated the risk score by summing the disease-related alleles, weighted by the odds ratio derived from an independent GWAS. Here, GWAS summary statistics of the enriched T2D risk alleles derived from the selected initial SNP list used as the discovery dataset. An r^2^ threshold of 0.1 and a window size of 250 kb were utilized for clumping the SNPs in linkage disequilibrium. Since the PRS is a continuous measure, we considered the quintiles of calculated PRS to categorize individuals as being at “low”, “moderate”, or “high” genetic risk groups. Next, the effect of the computed PRS on the T2D prevalence in the Iranian population was assessed by the robust Poisson regression analysis adjusted for the baseline BMI, sex, and age.

### Validation of the polygenic risk score with T2D incident analysis in Iranian population

The impact of the calculated PRS on T2D incidence was assessed in the individuals without the prevalent T2D at the baseline using Cox regression analysis. Time-to-event was calculated from the date of the baseline examination to the date of the first follow-up examination meeting our criteria for the T2D incidence; the date of the last examination was considered for each censored participant. The model is adjusted for age, sex, BMI, fasting plasma glucose, 2-h plasma glucose, triglycerides, total cholesterol, high-density lipoprotein cholesterol levels, and low-density lipoprotein cholesterol level at baseline.

### Association of the polygenic risk score with known biochemical T2D risk factors in Iranian population

The effect of PRS on fasting plasma glucose, 2-h plasma glucose, triglycerides, total cholesterol, high-density lipoprotein cholesterol levels, as well as low-density lipoprotein cholesterol level, was investigated in the individuals without prevalent T2D via the linear regression analysis. For each analysis, the model was adjusted for age, sex, BMI, and other biochemical T2D risk factors except for the dependent variable of interest under analysis.

## Supplementary information

Supplementary file1

Supplementary file2

Supplementary file3

Supplementary file4

## Data Availability

The data analyzed during the current study for the Iranian population are available from the corresponding author on reasonable request. The 1,000 Genome Project data used in the present study is publicly available at ftp://ftp.1000genomes.ebi.ac.uk/vol1/ftp/release/20130502/supporting/GRCh38_positions.
