# Supplementary material for: High genetic burden of type 2 diabetes can promote the high prevalence of disease: a longitudinal cohort study in Iran
Source: Sci Rep. 2020 Aug 19;10:14006. doi: 10.1038/s41598-020-70725-4 (PMC7438483; doi:10.1038/s41598-020-70725-4)
Supplement: Supplementary file 4 — Supplementary file4 [file 41598_2020_70725_MOESM4_ESM.pdf]

High genetic burden of type 2 diabetes can promote the high prevalence of disease: A longitudinal cohort study in Iran

Maryam Moazzam-Jazi, Leila Najd Hassan Bonab, Asiyeh Sadat Zahedi, Maryam S. Daneshpour*

This file includes the Supplementary Table 1. The other supplementary information is available as Excel format.

Supplementary Table 1. The association of PRS with fasting plasma glucose

Quintile	β (SE)	p-value
2 (20–40%)	0.2 (0.1)	0.63
3 (40–60%)	0.29 (0.12)	0.5
4 (60–80%)	0.7 (0.3)	0.09
5 (> 80%)	1.11 (0.32)	0.01

Model is adjusted for age, sex, BMI, 2-hour plasma glucose, cholesterol, triglyceride, high density lipoprotein cholesterol, and low density lipoprotein cholesterol levels. The first quintile (< 20%) was considered as reference.
